# Understanding the barriers and facilitators of healthcare services for brain injury and concurrent mental health and substance use issues: a qualitative study

**DOI:** 10.1186/s12913-024-11316-1

**Published:** 2024-08-02

**Authors:** Jasleen Grewal, Cole J. Kennedy, Rinni Mamman, Janelle Breese Biagioni, Mauricio A. Garcia-Barrera, Julia Schmidt

**Affiliations:** 1 University of British Columbia, Vancouver, BC Canada; 2 Rehabilitation Research Program, Centre for Aging SMART at Vancouver Coastal Health, Vancouver, BC Canada; 3 Department of Psychology, University of Victoria, Victoria, BC Canada; 4 Institute on Aging & Lifelong Health, University of Victoria, Victoria, BC Canada; 5 CGB Centre for Traumatic Life Losses, Victoria, BC Canada; 6 Department of Occupational Science & Occupational Therapy, University of British Columbia, Vancouver, BC Canada

**Keywords:** Acquired brain injury, Healthcare services, Patient perspectives, Qualitative

## Abstract

**Background:**

People with acquired brain injury (ABI) may experience concurrent conditions such as, mental health and substance use concerns, that require specialized care. There are services that aim to support people with ABI and these conditions separately; however, little is known about the facilitators and barriers of these services. Therefore, the purpose of this study was to engage stakeholders to investigate the facilitators and barriers of healthcare services for ABI and concurrent issues.

**Methods:**

Semi-structured focus groups were conducted in-person and virtually with people with ABI, caregivers, healthcare professionals, and policy makers during a one-day event in British Columbia, Canada. Manifest content analysis was used with a constructivist perspective to analyze data.

**Results:**

90 participants (including 34 people with ABI) provided insights during 15 simultaneous focus groups. Three categories were identified: (1) complexity of ABI, (2) supports, (3) structure of care. Complexity of ABI outlined the ongoing basic needs after ABI and highlighted the need for public awareness of ABI. Supports outlined healthcare professional and community-based supports. Structure of care described people with ABI needing to meet criteria for support, experiences of navigating through the system and necessity of integrated services.

**Conclusions:**

These findings highlight the facilitators and barriers of healthcare services for ABI and concurrent conditions and provide insights into the changes that may be needed. Doing so can improve the accessibility and quality of ABI healthcare services.

**Supplementary Information:**

The online version contains supplementary material available at 10.1186/s12913-024-11316-1.

## Introduction

Acquired brain injury (ABI) is a leading cause of disability worldwide [1, 2] comprising of both traumatic and non-traumatic subtypes [3–6]. Mental health and addiction-related issues, such as anxiety, depression and substance use disorders are common following ABI, and have been recognized as significant contributors to the morbidity in both types of ABI [7–10]. For example, approximately one-third of people with stroke experience post-stroke depression [11], and the risk of mental health disorders is increased two-fold following traumatic brain injury (TBI) [12]. Broadly, it is estimated that upwards of 60% of people with ABI struggle with a mental health or substance use disorder [5]. However, these statistics are largely based on data from hospitalized or treatment-seeking patients [12]. It is suggested that a much larger proportion of people with ABI experience challenges with mental health and substance use than what the current epidemiological rates suggest [13].

Health comorbidities such as mental health and substance use concerns are both a risk factor for ABI incidence and a commonly occurring outcome of ABI [6, 14, 15]. Regardless of time of onset, these mental health and substance use concerns are associated with lower quality of life, higher healthcare costs and decreased life expectancy after ABI [16, 17]. Current ABI services in British Columbia (BC), Canada within a publicly funded healthcare system, include hospital-based services (i.e., rehabilitation) right after injury. Depending on the severity of injury, some people are given outpatient rehabilitation and community support when discharged from hospital. If a person obtains the ABI due to work-related reasons or in a motor vehicle accident, they may have private coverage through insurance.

People with concurrent ABI, mental health disorders and substance use have unique treatment needs that remain largely unaddressed due to limited communication and coordination between healthcare services and service providers. Physicians, nurses, rehabilitation professionals and mental health supports are involved in a patient’s inpatient care [18]. Referrals may then be made for other healthcare professionals (i.e., case managers) to support the person’s critical transition from the hospital to the community [19]. However, an important caveat is the systemic reliance from the healthcare system on self-advocacy, such that the person needing care needs to seek it (e.g., identify clinics, make appointments) and receive it (e.g., remember the appointments, arrange transportation) themselves. As a result, impairments common to ABI, such as decreased self-awareness, self-efficacy and organizational abilities may make it difficult for people to access and engage with services [20, 21]. Memory-related and other neurocognitive challenges with treatment content and keeping track of materials has been identified as a barrier to care provision for people with TBI in psychological distress [22].

Integrated healthcare services can help meet the needs of people while building a more effective and efficient healthcare system [23]. Integrated care aims to provide holistic services for people that have complex and intersectional healthcare issues. Chan and colleagues’ (2022) systematic review summarized previous studies that discussed integrated intervention activities for TBI, mental health and substance use care. Studies reported barriers (e.g., lack of education) and/or facilitators (e.g., inclusion of family) to integrated ABI, mental health and substance use care. Of the studies exploring barriers or facilitators, only one used qualitative methods to understand stakeholders’ perspectives [21]. In this study, authors conducted qualitative interviews and focus groups to determine barriers (e.g., poor service provider education, limited access to care) and facilitators (e.g., patient education and caregiver support) to diagnose and treat neuropsychiatric disturbances following TBI. The authors suggested that improving quality of care may require a more comprehensive investigative approach, such as including people with both traumatic and non-traumatic brain injuries, focusing on a broader range of mental health disorders and addressing substance use challenges [24].

Of the studies included in Chan and colleagues’ (2022) systematic review, many (51%) focused on military service members, and none specifically focused on underserved or marginalized populations (e.g., homeless or marginally housed, Indigenous) in which ABI occurrence is disproportionally high, and long-term outcomes are poorer relative to the general population [25–27]. Marginalization and stigma may deter people from seeking care or disclosing their condition(s) to healthcare professionals, further exacerbating the fragmentation problem. Researchers and community advocates have called for greater inclusion of marginalized groups in ABI research to understand their treatment needs and barriers to healthcare services [13].

Research on the barriers and facilitators of ABI healthcare services is limited. Of the extant studies, most have focused on subtypes of ABI, while few have explored the intersections of mental health and substance use concerns. Moreover, studies have primarily focused on specific populations (e.g., military with experience of brain injury) or certain subgroups related to ABI (e.g., people with ABI and concurrent issues, healthcare professionals) in isolation, rather than leveraging their collective experience through collaboration. Importantly, there is a lack of meaningful inclusion of marginalized groups across the literature and limited use of partnered approaches to understand healthcare service delivery.

The purpose of the present study was to engage key stakeholders to identify barriers and facilitators to healthcare service delivery for people with concurrent ABI and mental health and/or substance use concerns.

## Methods

### Design

We conducted a qualitative study using phenomenological methodology to understand the experience, context and phenomenon of navigating through healthcare services for ABI and concurrent conditions [28]. Given the complexity of multiple types of participants, various focus groups and a large sample size, we chose to conduct manifest content analysis to understand a broad range of experiences, but only on the immediate, surface level elements of what people expressed. We obtained research ethics approval from the University of Victoria (#22–0614) and the University of British Columbia (#H22-03403). Findings are reported using the COnsolidated criteria for REporting Qualitative research (see Additional File 1) [29].

### Participants and context

Participants were purposively recruited through contacting ABI community associations, health authorities, healthcare organizations, community stakeholders and government representatives in-person or via email in BC to attend the Consensus on Brain Injury Day on October 14, 2022. This event was the first of a 3-year initiative to improve healthcare services for people with ABI and concurrent concerns (e.g., mental health, substance use, intimate partner violence and homelessness). The organizations identified potential participants that were involved with or affected by the intersections of ABI and mental health and/or substance use, including researchers, healthcare professionals, service providers, brain injury association representatives, policy makers, health administrators, and people with ABI and concurrent issues and their caregivers. Consent to participate was collected online through an invitation and survey. The survey asked demographic questions including which role they identified as (e.g., person with lived experience, researcher, etc.). Participants self-reported ABI diagnosis (if applicable) and/or their role in relation to ABI and mental health and/or substance use. Participants were explained the purpose of the event through the consent form or verbally if needed. To fill the current gap in the literature, we had targeted recruitment initiatives for minority groups, such as people with physical or intellectual disabilities, members of Indigenous groups and members of the LGBTQ2IAS + community, to identify culturally informed barriers and facilitators to healthcare services. People with lived experience were provided an honorarium for participation.

### Data collection and analysis

Data were collected during concurrent in-person and virtual (via Zoom) focus groups at the Consensus on Brain Injury Day. Participants were assigned to focus groups based on their role and experience, aiming to include diverse experiences and opinions in each focus group. For example, each focus group aimed to have a person with lived experience, a caregiver and a healthcare professional. The focus groups were conducted by 16 facilitators (13 females and three males) who had experience working with diverse and vulnerable populations including those affected by ABI. They were provided training materials and a 1-hour training focused on teaching how to manage group dynamics (i.e., redirecting conversation) and how to ensure all voices were heard during the conversations. The training also focused on managing own assumptions and biases when facilitating the focus groups. The facilitators included ten undergraduate and graduate students and six ABI service providers, who had no prior relationship with the participants. The Consensus on Brain Injury Day was also facilitated by an external professional facilitator who had no prior relationship with the participants. The organizing committee was present to assist the facilitators if needed.

The event started with speakers (i.e., researchers, clinicians, event organizers) relaying the intersection of ABI, mental health and substance use to orient participants to the topic. During this time, participants could engage in the conversation and ask questions. Then focus groups were conducted and asked three broad questions (see Additional File 2): (1) What is working well in healthcare service delivery for people with ABI and mental health and/or substance use? (2) What is not working well in healthcare service delivery for people with ABI and mental health and/or substance use? and (3) What is one thing I would change in healthcare service delivery for people with ABI and mental health and/or substance use? The questions aimed to understand the experience of participants on healthcare service delivery for people experiencing concurrent ABI, mental health and/or substance use concerns. To summarize data, the facilitators asked participants to choose one to three most important points for each question and these responses were recorded. Then facilitators confirmed if the recorded responses reflected the thoughts of all participants in the focus group. Once confirmation was received by participants, these summaries were provided to the organizing committee. These summarized responses were used in the analysis of this study, with quotes corresponding to the main points identified by each participant.

Manifest content analysis using a constructivist perspective was used to analyze the data [30]. Researchers JG (female, person of colour, occupational therapist, graduate student, experience with ABI population in clinical and research settings), RM (female, person of colour, graduate student, experience with ABI population in research setting) and JS (female, occupational therapist, assistant professor, experience with ABI population in clinical and research settings) read the summarized data to gain data immersion. JG then coded the data line-by-line on Microsoft Excel to capture similarities and differences. After initial coding, JG, RM and JS collaboratively categorized the data to generate categories. To ensure trustworthiness, the authors employed reflexivity by consistently meeting during data analysis to discuss personal biases and impact on data analysis [31]. The involvement of a heterogenous group of participants and multiple focus groups facilitators provided rich complementary perspectives on the topic. Conducting multiple focus groups allowed to hear mixed experiences and perspectives.

## Results

A total of 90 participants participated in this study during 15 concurrent focus groups. The mean age of participants was 47.2 years, with most participants (*n* = 63, 70%) being female. Five participants did not provide an answer. Amongst the 90 participants, 34 participants were people with lived experiences of ABI living with injury for a mean of 14.47 years. Other participants were family members supporting people with ABI (*n* = 14), service providers or healthcare professionals (*n* = 28), researchers (*n* = 10) and government representatives (*n* = 3). Participants also indicated other roles, such as a community advocate (*n* = 1), retired healthcare professional (*n* = 1) and others. Four participants did not provide an answer. A participant could identify as more than one role. See Table 1 for participant details.

**Table 1:** Demographic characteristics

Demographic categories	All participants (*n* = 90)
**Age** (*m* ± SD)	47.24 ± 15.22
**Years affected by ABI (***n*** = 34)** (*m* ± SD)	14.47 ± 9.73
**Sex ***n* (%)	
Female	63 (69)
Male	22 (24.44)
Prefer not to answer	2 (2.22)
**Gender ***n* (%)	
Female	64 (71.11)
Male	21 (23.33)
Non-binary or Indigenous Two-Spirit	2 (2.22)
**Sexual orientation ***n* (%)	
Heterosexual	70 (77.77)
Bisexual	6 (6.66)
Homosexual	4 (4.44)
Indigenous Two-Spirit	1 (1.11)
Not listed	2 (2.22)
Prefer not to answer	4 (4.44)
**Ethnicity ***n* (%)	
White	64 (71.11)
East Asian	5 (5.55)
Indigenous	3 (3.33)
South Asian	2 (2.22)
Indigenous and White	2 (2.22)
Black and East Asian	1 (1.11)
Black and White	1 (1.11)
East Asian and White	1 (1.11)
East African	1 (1.11)
White and Indo-Carribean	1 (1.11)
Other	4 (4.44)
Prefer not to answer	2 (2.22)
**Education ***n* (%)	
Bachelor’s degree	27 (30)
College diploma	20 (22.22)
Some post-secondary education	12 (13.33)
Master’s degree	9 (10)
Doctoral degree	9 (10)
High school diploma	6 (6.66)
Some high school	3 (3.33)
Less than high school	1 (1.11)

NOTE. Total *n* = 90, 3 participants did not consent to collection of demographic information

From the data, three categories were identified, “complexity of ABI”, “support” and “structure of care”. Subcategories were identified, relaying the benefits, challenges and needs of ABI healthcare services.

### Category 1: complexity of ABI

Participants emphasized the complexity of ABI, and this encompassed long-term, often unmet, basic needs after ABI and accompanying concurrent issues. “Public awareness of the complexity” and “ongoing basic needs after ABI” were the subcategories identified.

#### Subcategory 1a: ongoing basic needs after ABI

Participants described barriers to address basic needs, such as a lack of financial assistance for healthcare services after experiencing an ABI and concurrent concerns. Participants reflected that there were differences in financial situations depending on the cause of ABI (e.g., motor vehicle accident versus fall), with more financial security after some injuries due to funding through insurance. Participants noted, “there is a disparity in financials dependent on type of injury [for example] car accident injury versus intimate partner violence injury” (in-person, group 1).

The need for long-term funding for basic healthcare needs and housing support was emphasized after ABI and concurrent concerns. Participants described, “lack of long-term funding for supportive housing, outreach [or] community support, employment [or] volunteering, health supports, disability, dental, nutrition” (in-person, group 7). Participants highlighted system-level issues when accessing safe and affordable housing, such as the lack of funding assistance or subsidized housing agencies not having adequate units available:

“No affordable housing. [Disability assistance] is not enough to cover rent, bills, food. Shelter allowance of [dollar amount] per month is not enough. [Subsidized housing agency] doesn’t have enough units available. Can’t apply for rental assistance because you are already on [disability assistance]…” (virtual, group 5).

Participants also discussed the need for specialized housing for people with ABI and concurrent concerns, noting “A lack of housing, more housing [is needed] for people with brain injury and more specialized housing [for those] dealing with addiction issues and/or mental health concerns” (virtual, group 6).

#### Subcategory 1b: public awareness of the complexity

Participants indicated a need for awareness and education of ABI and concurrent issues in the public. Participants described a lack of awareness about the diverse symptoms and concurrent issues after ABI, and the likelihood of people experiencing an ‘invisible injury’ where it is not physically obvious that a person has an impairment. Participants also expressed the limited knowledge of the public, noting people tend to “view brain injury as an acute condition, rather than chronic” (in-person, group 5). Participants stated their behaviours may be criminalized due to the lack of understanding of ABI symptoms (i.e., associated mental health and substance use concerns). While some participants noted that public education on ABI has improved, others noted that there needs to be more education on the lifelong impacts of ABI.

Participants stated they experienced stigma often a result of insufficient education and awareness of ABI and concurrent concerns. Participants noted, “Not enough open education for  [the] general public, invisible nature [of brain injury] [leads to] stigma [in] employment and [not] disclosing to people” (virtual, group 4). Participants reported on ways to reduce stigma, which included the need for advocacy programs and improving education of ABI and concurrent issues in the public. For example, participants noted the need for  “community education nationally and provincially of brain injury and concurrent concerns for awareness… [to] dismantle stigma” (virtual, group 4).

### Category 2: supports

Participants reflected on the available supports after ABI and concurrent concerns, and strategies to improve support. “Healthcare professional support” and “community-based support” were the subcategories identified.

#### Subcategory 2a: Healthcare professional support

Participants described the importance of healthcare professional support, specifically stating the importance of healthcare professionals being knowledgeable in ABI and concurrent concerns and being committed to improving the system. Participants emphasized difficulty when accessing supports due to the lack of healthcare professionals, “[There are] no family doctors; when you don’t have a gatekeeper, all we have is a locked gate” (virtual, group 1). Participants described that accessing specialized care was also difficult after ABI and concurrent concerns, and having a diagnosis was essential to receive services, “A lack of primary and follow-up care… general practitioners are not adequately trained to assess mental health and cognitive disability. [They are] hesitant to diagnose traumatic brain injury which limits access to services” (virtual, group 3).

Participants stated that healthcare professionals may lack understanding of the complexities of ABI and concurrent concerns, and require training to provide trauma-informed care, noting, “There is a lack of understanding and awareness for the different needs of brain injury survivors. More education is needed for medical [staff], first responders…” (virtual, group 6). Additionally, participants described there is a lack of continuous care due to changing healthcare professionals during their care. To counteract this, participants emphasized the importance of staff retention, through better wages and incentives, for continuous care. At times, due to healthcare professionals’ lack of understanding of ABI, participants expressed they experienced stigma when accessing healthcare services, especially if there were concurrent mental health and substance use concerns. Participants reflected that these types of experiences may be emotionally distressing for people with ABI and their caregivers, “Stigma, especially in health[care] services creates harm and dangerous situation for individuals and families” (virtual, group 1). Many participants indicated the need to reduce stigma in healthcare and the public by improving education for ABI and concurrent issues, including recognition in high-risk situations (e.g., substance use, homelessness, intimate partner violence).

Secured funding in the healthcare system was emphasized by participants. Participants described a lack of funding available which could impact the availability and accessibility of specialized services, “Services cut due to lack of funding. Services rely on individual advocacy from staff and if [they are] not there, they are cut without input from those who utilize them…” (virtual, group 3). Participants stated there needs to be increased funding that can improve staffing and service availability, noting “Opportunity for funding for services, collaboration between service providers, securing funding from provincial government. Allows consistent staffing and specialized programming; continue to lobby the provincial government for funding…” (virtual, group 3).

#### Subcategory 2b: community-based support

Participants relayed the importance of needing support from community-based supports, such as needing home-based support from interdisciplinary rehabilitation professionals. This was particularly noted to be important if concurrent concerns were present due to the added difficulty in adjusting to life at home after injury. Participants noted, “[We need] initial transition to home care by a multidisciplinary team [such as] physiotherapy, occupational therapy and speech therapy” (virtual, group 3). However, participants highlighted that these supports may be limited, describing “Front end of rehabilitation [is] usually well-funded for initial weeks [to] months until they are ready to go home. Downside is for continuity of care and long-term transition from inpatient to outpatient” (virtual, group 3). Along with outpatient rehabilitation support, having a case manager and support for daily needs were described to be important to improve the ability to re-integrate into their lives and live independently, especially if a person experienced mental health concerns. Participants emphasized the importance of receiving support to develop skills and meet goals after ABI, describing a “need [for] mandated incentives [or] supports [for] community engagement [and] life skills…” (in-person, group 7). Participants stated there were limited support for families of people with ABI, which is a gap in healthcare service delivery. Participants highlighted the need for collaboration between community organizations and resources for service navigation (e.g., documents summarizing services) as benefits.

Participants described wanting to feel like they belong to a community through peer support and hearing others’ lived experiences. Likewise, participants described that when concurrent concerns were present, having a community worker enhanced engagement in daily activities, stating “having a consistent mental health worker who meets me where I am at, helps me with all daily activities [such as] parenting, eating, or to chat…” (in-person, group 6). Ultimately, participants emphasized that being asked what they need and being seen as a human (versus their diagnoses) helped their sense of belonging.

### Category 3: structure of care

Participants talked about accessing and navigating healthcare services after ABI and concurrent concerns and reflected on the integration of ABI healthcare services. “Meeting criteria for support”, “navigating through the system” and “integrating services” were the subcategories identified.

#### Subcategory 3a: meeting criteria for support

Participants described the system-level barriers to meet criteria to obtain support after ABI and concurrent issues. For example, participants stated concerns with the medical system and obtaining a diagnosis, describing the “medical system, [as the] first point of contact [is] not effective. Diagnosis, appreciating the complexity of people that are accessing physicians. Hospital [is] often where people are being denied services…” (virtual, group 5). Participants stressed the importance of a prompt diagnosis of ABI and concurrent issues to obtain supports in a timely manner. Apart from the diagnosis, participants reflected there was a need for referrals to access care, and inclusion and exclusion criteria to obtain support which made it difficult to qualify for supports. This was especially important if participants wanted to seek specialized supports for the concurrent concerns they were experiencing. Other systemic barriers to receiving services included long wait times to receive care, with participants reporting “long wait periods for essential services create a sense of doom, need multiple referrals and appointments to get the necessary specialized care for ABI and concurrent issues” (in-person, group 6).

Participants emphasized that to receive services, documentation may be required, which may be difficult to complete due to the symptoms of ABI and concurrent concerns. Additionally, missing appointments, often due to ABI and concurrent concerns, and subsequently being charged for missed appointments were a barrier when accessing supports. Participants also described that geographical location impacted access to support, “inequitable access to services on the basis of geographic location and cost” (in-person, group 5), and stressed needing local access to supports.

#### Subcategory 3b: navigating through the system

Participants outlined that availability to support was important. Participants stated that there were not enough specialized supports available after experiencing an ABI and concurrent concerns. Participants also noted a lack of knowledge of available resources and being unsure how to access resources. Participants described specific barriers to access supports, such as “scattered services, difficult to navigate, lack of communication” (virtual, group 1). Other participants stated a lack of prioritization of services to help navigate supports, “navigation in a broken system. Currently done off the side of someone’s desk, needs to be someone’s full role/position on its own” (virtual, group 5). This resulted in participants and their families feeling stressed. Participants working in healthcare also indicated difficulties with navigating supports, stating “working within healthcare, [it is] difficult to navigate services to best support patients with ABI [or] mental health, addictions… complex care” (virtual, group 1).

To improve access to supports, participants reflected that there needs to be one place to access all kinds of supports after experiencing an ABI and concurrent concerns. For instance, participants emphasized, “[need] changing [of] [current healthcare model], [there is] information disconnect [and] procedural disconnect. [Need] easy access [to] streamlined services, one hub” (in-person, group 8). Participants reflected that a hub approach (i.e., one place to access supports) was currently working well in areas of BC and if implemented widely, this may improve continuity of care. Participants proposed needing one place to access resources, highlighting “One pager for patients [and] families of treatment [or] housing options. They should not have to navigate through the internet. Pathway to help navigate” (virtual, group 4).

#### Subcategory 3c: integrating services

Participants emphasized the need for continuous and integrated services for people with ABI and concurrent conditions. Participants stressed there needs to be coordination between programs to ensure continuity between programs. Participants stated, “there is often siloed care [and] disconnection between programs and understanding of brain injury and concurrent issues” (virtual, group 1). To counteract this, participants emphasized the importance of implementing a targeted pathway of care for integrated care. Additionally, participants stated that integrated care could be enhanced by implementing care comprising of interdisciplinary healthcare professionals. Participants reflected that integration of services could facilitate improved communication between service providers, “It is important to make it easier for practitioners to connect with one another regarding clients, making it easier for interdisciplinary care and preventing [the] client being forced to repeat information” (in-person, group 6).

Participants highlighted the need for holistic and individualized services, noting the complexity of ABI and the additional diagnoses that people may have. Participants stated there needs to be improved “understanding of the holistic scope [or] lens needed for brain injury recovery” (in-person, group 5). Likewise, participants stressed the importance of needing specialized programs and supports after ABI and concurrent conditions, such as access to legal services and mental health support, “Mental health services don’t take into account individuals with brain injury, no combined care between brain injury and mental health [or] no specialized area [or] support for [people] with brain injury and mental health [concerns]” (in-person, group 4).

## Discussion

Our study identified the perceived barriers, facilitators and suggestions to improve the quality of ABI healthcare services for people with concurrent issues from the perspectives of multiple stakeholders in BC. These findings demonstrate the complexity of ABI and the requirement for ongoing basic needs and funding provision. Our data aligns with previous research that states that there is a lack of funding for ABI programs in other areas of Canada (i.e., Ontario) [32]. Similar reports were found in the United Kingdom [33] and United States [34]. Like previous research, our study identified that funding may be improved for people with ABI who are injured at work or during motor vehicle accidents, through access to third-party insurance versus government funding [35–37]. Past research also indicates that third-party insurance companies often have poor understanding of ABI consequences and concurrent diagnoses, therefore the funding provision even with insurance is not consistent [34].

We identified participants reported a lack of public awareness and education that hindered their experience of healthcare service delivery which is supported by previous research [38]. Public education programs may be helpful to improve awareness and knowledge of ABI and concurrent issues [39]. However, some studies have shown no positive effects of education programs alone on the knowledge of ABI [40, 41].

Our results speak to the challenges of accessing healthcare professional support, especially limited access to general physicians, which results in restricted referrals to specialized ABI supports and services. This is a widespread issue across Canada, with research showing that one in seven Canadians do not regularly access general physicians [42] even though regular access can result in timely care [43] and improved patient satisfaction [44]. This dilemma is likely due to shortages of general physicians in Canada, with BC, amongst other provinces, having the fewest general practitioners per 100,000 persons [45]. Notably, when people need to access specialist physicians (i.e., psychiatrists) to address concurrent conditions, wait times are significant and worsening in the recent years [46, 47]. Additionally, there can be misconceptions and prejudices when accessing care, for instance healthcare professionals may have misconceptions of the person’s abilities if a person has an ‘invisible’ ABI [38]. Even when people have access to healthcare services, healthcare professionals often address the physical symptoms resulting from ABI and less attention is given to the associated psychological concerns [47]. A review emphasized that people with ABI along with their caregivers require professional psychological support [48]. To exacerbate this gap in service, healthcare professionals may have prejudicial attitudes and reduced offering of support if they feel people are responsible for their ABI [49]. Similarly, reviews have shown that healthcare professionals may have stigma towards people with psychiatric illnesses or substance use concerns and this may result in suboptimal care [50, 51]. Therefore, it is unsurprising that if a person experiences an ABI and mental health and/or substance use concerns, they may experience stigma from healthcare professionals, and this can result in reduced care. It is important to implement education (e.g., multidisciplinary team meetings) for healthcare professionals to understand ABI symptoms and concurrent conditions to help change perception and thus improve interactions between people with ABI and healthcare professionals [24, 52].

Our results highlight the importance of interdisciplinary supports for people with ABI with associated mental health and substance use concerns, which is supported by previous research [24]. Interdisciplinary supports may involve support from case managers who provide individualized and collaborative supports to enhance skill acquisition and independent living after ABI [53, 54]. Additionally, community-based supports were identified to be important which are shown to be effective at meeting the long-term needs of people with ABI [55] by providing informational, psychosocial and self-management supports [33, 56, 57]. Community-based supports may also help people with ABI connect to peers which is beneficial for the adjustment period after ABI [58, 59].

Our results examine the system-level barriers to accessing healthcare services, such as meeting criteria of support. System-level barriers included needing referrals to access support which is impacted by patient (e.g., severity of injury, social and demographic factors), contextual (e.g., involvement of rehabilitation professionals, place of treatment) and decision-making factors (e.g., healthcare professionals considering all factors) [60]. Early referral programs may be beneficial as they are seen to improve social integration, emotional well-being and vocational functioning [61]. People with ABI may also not receive a timely diagnosis, especially if the injury is mild [62], limiting their access to services.

To improve access to care, our study emphasized the importance of improved accessibility to supports by being cognizant of which services are available and having assistance for service navigation. A study showed that when people with ABI are assigned a dedicated case worker to connect them to resources and help them navigate the healthcare system, their community integration, independence and functional abilities were improved [63]. Similarly, with other diagnoses (e.g., cancer), patient navigation has shown to provide timely access to care and provide support through the recovery journey [64, 65]. Importantly, our results highlight that integrated care is essential when receiving ABI healthcare services. Past research has shown that integrated care can improve functional outcomes and reduce the length of stay for people with ABI [66]. Additionally, the systematic review by Chan et al. (2022) identified that integrated care for people with TBI and concurrent mental health and substance use concerns at the clinical level (i.e., professionals providing care) can result in improved outcomes (e.g., reduced symptoms and improved participation). In this study, lack of education, limited access to care, healthcare providers’ hesitancy and difficulties with technology were recognized as barriers to integrated care, while identified facilitators were inclusion of family or caregivers in the treatment process and compensatory strategies for cognitive challenges [67]. Further, integration at the meso-level may result in including a multidisciplinary team of healthcare professionals to address various impairments and symptoms. Multidisciplinary teams can enhance education amongst professionals to improve ability to diagnose and reduce healthcare professionals’ perceived lack of experience [68, 69]. Finally, having one place to access services can reduce travel-related barriers which may improve access to care [68]. Integrated care may be beneficial for ABI healthcare services; however, further research is needed to understand how to best design and implement this type of care with ABI and concurrent conditions.

### Limitations

Our study had six main limitations. First, we recruited participants who had connections with ABI associations in BC, known healthcare professionals and community advocates. This may have limited the perspectives represented within our results, such that our sample may not reflect the perspectives of those who are less connected with services. However, we purposively sought to include representatives from commonly marginalized and excluded groups (e.g., indigenous, LGBTQ2IAS+) to obtain perspectives from multiple and heterogenous groups, providing a breadth of experiences and opinions. Second, some participants may have not been available to participate due to having other commitments (i.e., work). To ensure participant availability, we provided an honorarium for people with lived experience to avoid their loss of income. We also provided the option of attending virtually if participants had other commitments. For future events, it may be helpful to schedule the focus groups during lunch breaks, after work or on a weekend to improve attendance of working individuals. Third, participants with ABI may have had challenges in participating in the event, limiting the quality of the responses. For example, we included participants with ABI who could participate in focus groups during the Consensus on Brain Injury Day event; therefore, people with severe communication challenges did not participate. However, we provided the option to engage virtually if this was more suitable to participants. Fourth, we recognize that facilitators may have had their own biases during focus groups. We mitigated this through providing a training session for facilitators. Next, participating in the full event may have caused fatigue for some participants with ABI and thus reduced their level of engagement. However, we offered multiple breaks, assistance and accommodations for people with different challenges. Lastly, data were collected through summaries of participant discussions and not through direct recording and transcribing of participant responses, therefore some data may have been misinterpreted or not fully accounted. However, the summarized data provides a variety of experiences and responses from a large sample, which helps to inform how ABI service delivery can be enhanced.

## Conclusion

Findings from this study provide insight into the barriers, facilitators and needs of ABI healthcare service delivery in BC including a lack of public awareness and knowledge of ABI and concurrent concerns, the importance of healthcare professional and community support, and the need for integrated care after ABI and concurrent concerns. These findings may help improve access to ABI healthcare services and can enhance the quality of support after ABI and concurrent concerns. Future research should strive to leverage these findings by investigating ways to break down barriers and enhance facilitators to improve ABI healthcare service provision in BC and abroad.

### Electronic supplementary material

Below is the link to the electronic supplementary material.

Supplementary Material 1

Supplementary Material 2

## Data Availability

The datasets used and/or analyzed during the current study are available from the corresponding author on reasonable request.

## Abbreviations

ABI: Acquired brain injury
BC: British Columbia
TBI: Traumatic brain injury

## References

[1] CDC. Traumatic brain injury in the United States. 2002. Available from: www.cdc.gov/TraumaticBrainInjury.

[2] Brain Injury Canada. https://www.braininjurycanada.ca/en/statistics-brain-injury. 2022. Statistics on brain injury.

[3] Ackermark PY, Schepers VP, Post MW, Rinkel GJ, Passier PE, Visser-Meily JM. Longitudinal course of depressive symptoms and anxiety after aneurysmal subarachnoid hemorrhage. Eur J Phys Rehabil Med. 2017;53(1). 10.23736/S1973-9087.16.04202-7 27412071

[4] Almeida OP, Hankey GJ, Yeap BB, Golledge J, Flicker L. Prevalence, associated factors, mood and cognitive outcomes of traumatic brain injury in later life: the health in men study (HIMS). Int J Geriatr Psychiatry. 2015;30(12):1215–23. 25703581 10.1002/gps.4276

[5] Kim E, Lauterbach EC, Reeve A, Arciniegas DB, Coburn KL, Mendez MF, et al. Neuropsychiatric complications of traumatic brain injury. J Neuropsychiatry Clin Neurosci. 2007;19(2):106–27. 17431056 10.1176/jnp.2007.19.2.106

[6] Ponsford J, Alway Y, Gould KR. Epidemiology and natural history of psychiatric disorders after TBI. J Neuropsychiatry Clin Neurosci. 2018;30(4):262–70. 29939106 10.1176/appi.neuropsych.18040093

[7] Ilie G, Mann RE, Hamilton H, Adlaf EM, Boak A, Asbridge M, et al. Substance use and related harms among adolescents with and without traumatic brain injury. J Head Trauma Rehabilitation. 2015;30(5):293–301. 10.1097/HTR.0000000000000101 25427256

[8] Ilie G, Adlaf EM, Mann RE, Ialomiteanu A, Hamilton H, Rehm J, et al. Associations between a history of traumatic brain injuries and current cigarette smoking, substance use, and elevated psychological distress in a population sample of Canadian adults. J Neurotrauma. 2015;32(14):1130–4. 25496189 10.1089/neu.2014.3619 PMC4504340

[9] Chemerinski E, Robinson RG. The neuropsychiatry of stroke. Psychosomatics. 2000;41(1):5–14. 10665263 10.1016/S0033-3182(00)71168-6

[10] Sloan MA, Kittner SJ, Rigamonti D, Price TR. Occurrence of stroke associated with use/abuse of drugs. Neurology. 1991;41(9):1358–1358. 1891081 10.1212/WNL.41.9.1358

[11] Hackett ML, Yapa C, Parag V, Anderson CS. Frequency of depression after stroke. Stroke. 2005;36(6):1330–40. 15879342 10.1161/01.STR.0000165928.19135.35

[12] Perry DC, Sturm VE, Peterson MJ, Pieper CF, Bullock T, Boeve BF, et al. Association of traumatic brain injury with subsequent neurological and psychiatric disease: a meta-analysis. J Neurosurg. 2016;124(2):511–26. 26315003 10.3171/2015.2.JNS14503 PMC4751029

[13] BC Consensus on Brain Injury. BC consensus statement on brain injury, mental health & addiction. 2023.

[14] Gould KR, Ponsford JL, Johnston L, Schönberger M. The nature, frequency and course of psychiatric disorders in the first year after traumatic brain injury: a prospective study. Psychol Med. 2011;41(10):2099–109. 21477420 10.1017/S003329171100033X

[15] Kureshi N, Clarke DB, Feng C. Association between traumatic brain injury and mental health care utilization: evidence from the Canadian community health survey. Inj Epidemiol. 2023;10(1):16. 36915175 10.1186/s40621-023-00424-x PMC10012583

[16] Hanafy S, Xiong C, Chan V, Sutton M, Escobar M, Colantonio A et al. Comorbidity in traumatic brain injury and functional outcomes: a systematic review. Eur J Phys Rehabil Med. 2021;57(4). 10.23736/S1973-9087.21.06491-1 PMC10396401 33541041

[17] Harrison-Felix C, Pretz C, Hammond FM, Cuthbert JP, Bell J, Corrigan J, et al. Life expectancy after inpatient rehabilitation for traumatic brain injury in the United States. J Neurotrauma. 2015;32(23):1893–901. 25057965 10.1089/neu.2014.3353 PMC6082166

[18] Turner-Stokes L. Evidence for the effectiveness of multi-disciplinary rehabilitation following acquired brain injury: a synthesis of two systematic approaches. J Rehabil Med. 2008;40(9):691–701. 18843419 10.2340/16501977-0265

[19] Curran C, Dorstyn D, Polychronis C, Denson L. Functional outcomes of community-based brain injury rehabilitation clients. Brain Inj. 2015;29(1):25–32. 25180709 10.3109/02699052.2014.948067

[20] Hunt AW, Turner GR, Polatajko H, Bottari C, Dawson DR. Executive function, self-regulation and attribution in acquired brain injury: a scoping review. Neuropsychol Rehabil. 2013;23(6):914–32. 24032652 10.1080/09602011.2013.835739

[21] McAllister TW. Neurobehavioral sequelae of traumatic brain injury: evaluation and management. World Psychiatry. 2008;7(1):3–10. 18458777 10.1002/j.2051-5545.2008.tb00139.x PMC2327235

[22] Dindo L, Johnson AL, Lang B, Rodrigues M, Martin L, Jorge R. Development and evaluation of an 1-day acceptance and commitment therapy workshop for veterans with comorbid chronic pain, TBI, and psychological distress: outcomes from a pilot study. Contemp Clin Trials. 2020;90:105954. 32032736 10.1016/j.cct.2020.105954 PMC8830556

[23] Suter E, Oelke N, Adair C, Armitage G. Ten key principles for successful health systems integration. Healthc Q. 2009;13(sp):16–23. 20057244 10.12927/hcq.2009.21092 PMC3004930

[24] Chan V, Toccalino D, Omar S, Shah R, Colantonio A. A systematic review on integrated care for traumatic brain injury, mental health, and substance use. PLoS ONE. 2022;17(3):e0264116. 35239715 10.1371/journal.pone.0264116 PMC8893633

[25] Keightley ML, Ratnayake R, Minore B, Katt M, Cameron A, White R, et al. Rehabilitation challenges for aboriginal clients recovering from brain injury: a qualitative study engaging health care practitioners. Brain Inj. 2009;23(3):250–61. 19205962 10.1080/02699050902748331

[26] Lasry O, Dudley RW, Fuhrer R, Torrie J, Carlin R, Marcoux J. Traumatic brain injury in a rural indigenous population in Canada: a community-based approach to surveillance. CMAJ Open. 2016;4(2):E249–59. 27398371 10.9778/cmajo.20150105 PMC4933602

[27] Stubbs JL, Thornton AE, Sevick JM, Silverberg ND, Barr AM, Honer WG, et al. Traumatic brain injury in homeless and marginally housed individuals: a systematic review and meta-analysis. Lancet Public Health. 2020;5(1):e19–32. 31806487 10.1016/S2468-2667(19)30188-4

[28] Lopez KA, Willis DG. Descriptive versus interpretive phenomenology: their contributions to nursing knowledge. Qual Health Res. 2004;14(5):726–35. 15107174 10.1177/1049732304263638

[29] Tong A, Sainsbury P, Craig J. Consolidated criteria for reporting qualitative research (COREQ): a 32-item checklist for interviews and focus groups. Int J Qual Health Care. 2007;19(6):349–57. 17872937 10.1093/intqhc/mzm042

[30] Downe-Wamboldt B. Content analysis: method, applications, and issues. Health Care Women Int. 1992;13(3):313–21. 1399871 10.1080/07399339209516006

[31] Morrow SL. Quality and trustworthiness in qualitative research in counseling psychology. J Couns Psychol. 2005;52(2):250–60. 10.1037/0022-0167.52.2.250

[32] Gan C, Gargaro J, Brandys C, Gerber G, Boschen K. Family caregivers’ support needs after brain injury: a synthesis of perspectives from caregivers, programs, and researchers. NeuroRehabilitation. 2010;27(1):5–18. 20634597 10.3233/NRE-2010-0577

[33] Odumuyiwa T, Kennedy M, Norman A, Holloway M, Suffield F, Forrest H, et al. Improving access to social care services following acquired brain injury: a needs analysis. J Long-Term Care. 2019;0(2019):164. 10.31389/jltc.6

[34] Meixner C, O’Donoghue CR, Witt M. Accessing crisis intervention services after brain injury: a mixed methods study. Rehabil Psychol. 2013;58(4):377–85. 24128269 10.1037/a0033892

[35] Mellick D, Gerhart KA, Whiteneck GG. Understanding outcomes based on the post-acute hospitalization pathways followed by persons with traumatic brain injury. Brain Inj. 2003;17(1):55–71. 12519648 10.1080/0269905021000010159

[36] Turner BJ, Fleming J, Ownsworth T, Cornwell P. Perceived service and support needs during transition from hospital to home following acquired brain injury. Disabil Rehabil. 2011;33(10):818–29. 20812814 10.3109/09638288.2010.513422

[37] Harrington R, Foster M, Fleming J. Experiences of pathways, outcomes and choice after severe traumatic brain injury under no-fault versus fault-based motor accident insurance. Brain Inj. 2015;29(13–14):1561–71. 26382715 10.3109/02699052.2015.1075142

[38] Swift TL, Wilson SL. Misconceptions about brain injury among the general public and non-expert health professionals: an exploratory study. Brain Inj. 2001;15(2):149–65. 11260765 10.1080/026990501458380

[39] Garcia JM, Sellers DM, Hilgendorf AE, Burnett DL. Evaluation of a health education programme about traumatic brain injury. Health Educ J. 2014;73(5):588–99. 10.1177/0017896913510512

[40] Hooper SR. Myths and misconceptions about traumatic brain injury: endorsements by school psychologists. Exceptionality. 2006;14(3):171–82. 10.1207/s15327035ex1403_5

[41] Linden MA, Braiden HJ, Miller S. Educational professionals’ understanding of childhood traumatic brain injury. Brain Inj. 2013;27(1):92–102. 23252440 10.3109/02699052.2012.722262

[42] Talbot Y, Fuller-Thomson E, Tudiver F, Habib Y, McIsaac WJ. Canadians without regular medical doctors. Who are they? Can Fam Physician. 2001;47:58–64. 11212435 PMC2014709

[43] Hayward RA, Bernard AM, Freeman HE, Corey CR. Regular source of ambulatory care and access to health services. Am J Public Health. 1991;81(4):434–8. 2003619 10.2105/AJPH.81.4.434 PMC1405059

[44] Fan VS, Burman M, McDonell MB, Fihn SD. Continuity of care and other determinants of patient satisfaction with primary care. J Gen Intern Med. 2005;20(3):226–33. 15836525 10.1111/j.1525-1497.2005.40135.x PMC1490082

[45] Li K, Frumkin A, Bi WG, Magrill J, Newton C. Biopsy of Canada’s family physician shortage. Fam Med Community Health. 2023;11(2):e002236. 37173094 10.1136/fmch-2023-002236 PMC10186392

[46] Liddy C, Moroz I, Affleck E, Boulay E, Cook S, Crowe L, et al. How long are canadians waiting to access specialty care? Retrospective study from a primary care perspective. Can Fam Physician. 2020;66(6):434–44. 32532727 PMC7292524

[47] Martinsen R, Kirkevold M, Sveen U. Young and midlife stroke survivors’ experiences with the health services and long-term follow-up needs. J Neurosci Nurs. 2015;47(1):27–35. 25565592 10.1097/JNN.0000000000000107

[48] Norman A, Curro V, Holloway M, Percuklievska N, Ferrario H. Experiences of individuals with acquired brain injury and their families interacting with community services: a systematic scoping review. Disabil Rehabil. 2023;45(4):739–51. 35244507 10.1080/09638288.2022.2043465

[49] Redpath SJ, Williams WH, Hanna D, Linden MA, Yates P, Harris A. Healthcare professionals’ attitudes towards traumatic brain injury (TBI): the influence of profession, experience, aetiology and blame on prejudice towards survivors of brain injury. Brain Inj. 2010;24(6):802–11. 20455671 10.3109/02699051003709623

[50] van Boekel LC, Brouwers EPM, van Weeghel J, Garretsen HFL. Stigma among health professionals towards patients with substance use disorders and its consequences for healthcare delivery: systematic review. Drug Alcohol Depend. 2013;131(1–2):23–35. 23490450 10.1016/j.drugalcdep.2013.02.018

[51] Henderson C, Noblett J, Parke H, Clement S, Caffrey A, Gale-Grant O, et al. Mental health-related stigma in health care and mental health-care settings. Lancet Psychiatry. 2014;1(6):467–82. 26361202 10.1016/S2215-0366(14)00023-6

[52] Dams-O’Connor K, Landau A, Hoffman J, St De Lore J. Patient perspectives on quality and access to healthcare after brain injury. Brain Inj. 2018;32(4):431–41. 29388840 10.1080/02699052.2018.1429024

[53] Tahan HA, Sminkey PV. Motivational interviewing. Prof Case Manag. 2012;17(4):164–72. 22660338 10.1097/NCM.0b013e318253f029

[54] McCluskey A, Johnson M, Tate R. The process of care management following brain injury: a grounded theory study. Brain Impairment. 2007;8(3):293–311. 10.1375/brim.8.3.293

[55] Fraas M, Balz M, DeGrauw W. Meeting the long-term needs of adults with acquired brain injury through community-based programming. Brain Inj. 2007;21(12):1267–81. 18236202 10.1080/02699050701721794

[56] Norman A, Holloway M, Odumuyiwa T, Kennedy M, Forrest H, Suffield F, et al. Accepting what we do not know: a need to improve professional understanding of brain injury in the UK. Health Soc Care Community. 2020;28(6):2037–49. 32364294 10.1111/hsc.13015

[57] Alhasani R, Radman D, Auger C, Lamontagne A, Ahmed S. Perspectives of clinicians and survivors on the continuity of service provision during rehabilitation after acquired brain injury. PLoS ONE. 2023;18(4):e0284375. 37043494 10.1371/journal.pone.0284375 PMC10096466

[58] Hughes R, Fleming P, Henshall L. Peer support groups after acquired brain injury: a systematic review. Brain Inj. 2020;34(7):847–56. 32421382 10.1080/02699052.2020.1762002

[59] Hibbard MR, Cantor J, Charatz H, Rosenthal R, Ashman T, Gundersen N, et al. Peer support in the community. J Head Trauma Rehabilitation. 2002;17(2):112–31. 10.1097/00001199-200204000-00004 11909510

[60] Foster M, Tilse C. Referral to rehabilitation following traumatic brain injury: a model for understanding inequities in access. Soc Sci Med. 2003;56(10):2201–10. 12697208 10.1016/S0277-9536(02)00236-8

[61] Reid-Arndt SA, Schopp L, Brenneke L, Johnstone B, Poole AD. Evaluation of the traumatic brain injury early referral programme in Missouri. Brain Inj. 2007;21(12):1295–302. 17963093 10.1080/02699050701721802

[62] Schmid KE, Tortella FC. The diagnosis of traumatic brain injury on the battlefield. Front Neurol. 2012;3. 10.3389/fneur.2012.00090 PMC3373009 22701447

[63] Rosario ER, Espinoza L, Kaplan S, Khonsari S, Thurndyke E, Bustos M, et al. Patient navigation for traumatic brain injury promotes community re-integration and reduces re-hospitalizations. Brain Inj. 2017;31(10):1340–7. 28650255 10.1080/02699052.2017.1325937

[64] Plant N, Mallitt KA, Kelly PJ, Usherwood T, Gillespie J, Boyages S, et al. Implementation and effectiveness of care navigation, coordinated management for people with complex chronic illness: rationale and methods of a randomised controlled trial. BMC Health Serv Res. 2013;13(1):164. 23642145 10.1186/1472-6963-13-164 PMC3645952

[65] Robinson-White S, Conroy B, Slavish KH, Rosenzweig M. Patient navigation in breast cancer. Cancer Nurs. 2010;33(2):127–40. 20142736 10.1097/NCC.0b013e3181c40401

[66] Laver K, Lannin NA, Bragge P, Hunter P, Holland AE, Tavender E, et al. Organising health care services for people with an acquired brain injury: an overview of systematic reviews and randomised controlled trials. BMC Health Serv Res. 2014;14(1):397. 25228157 10.1186/1472-6963-14-397 PMC4263199

[67] Janak JC, Cooper DB, Bowles AO, Alamgir AH, Cooper SP, Gabriel KP, et al. Completion of multidisciplinary treatment for persistent postconcussive symptoms is associated with reduced symptom burden. J Head Trauma Rehabilitation. 2017;32(1):1–15. 10.1097/HTR.0000000000000202 26709579

[68] Ahmed F, Bechtold K, Smith G, Roy D, Everett A, Rao V. Program of enhanced psychiatric services for patients with brain injury and neuropsychiatric disturbances: a proposed model of care. J Neuropsychiatry Clin Neurosci. 2016;28(2):147–52. 27093382 10.1176/appi.neuropsych.15060147

[69] Baig MR, Tapia RN, Meraj A, Pugh JA, Roache JD, Finley EP. Enhancing access to psychiatric care for posttraumatic stress disorder in veterans with mild traumatic brain injury through integrated services. Psychiatr Q. 2019;90(4):815–27. 31446544 10.1007/s11126-019-09668-7

