# Supplementary material for: Understanding the barriers and facilitators of healthcare services for brain injury and concurrent mental health and substance use issues: a qualitative study
Source: BMC Health Serv Res. 2024 Aug 2;24:881. doi: 10.1186/s12913-024-11316-1 (PMC11295555; doi:10.1186/s12913-024-11316-1)
Supplement: Supplementary file 2 — Supplementary Material 2 [file 12913_2024_11316_MOESM2_ESM.docx]

Interview Guide

1. What is working well in healthcare service delivery for people with ABI and mental health and/or substance use?
2. What is not working well in healthcare service delivery for people with ABI and mental health and/or substance use?
3. What is one thing I would change in healthcare service delivery for people with ABI and mental health and/or substance use?
